# Multi-objective optimization of cable-road layouts in smart forestry

**DOI:** 10.1080/14942119.2024.2380229

**Published:** 2024-08-11

**Authors:** Carl O. Retzlaff, Christoph Gollob, Arne Nothdurft, Karl Stampfer, Andreas Holzinger

**Affiliations:** a Human-Centered AI Lab, Institute of Forest Engineering, Department of Forest and Soil Sciences, University of Natural Resources and Life Sciences, Vienna, Austria; b Institute of Forest Growth, Department of Forest and Soil Sciences, University of Natural Resources and Life Sciences, Vienna, Austria

**Keywords:** Non-linear optimization, cable yarding, smart forestry, timber harvesting, layouts

## Abstract

Current cable-road layouts for timber harvesting in steep terrain are often based on either manual planning or automated layouts generated from low-resolution GIS data, limiting potential benefits and informed decision-making. In this paper, we present a novel approach to improve cable-road design using multi-objective optimization based on realistic cable-road representations. We systematically compare the effectiveness of single-objective and multi-objective optimization methods for generating layouts using these representations. We implement and evaluate the performance of a weighted single-objective approach, the AUGMECON2 and NSGA-II multi-objective methods in comparison to a layout manually created by a forestry expert, taking into account installation costs, harvesting volumes, residual stand damage and lateral yarding workload. In addition to implementing the first linear programming multi-objective optimization for realistic cable-road representations by adapting AUGMECON2, we also present the first implementation of a multi-objective genetic algorithm (NSGA-II) with simulated annealing for this purpose and evaluate their respective strengths. We find that the use of multi-objective optimization provides advantages in terms of cost-effective, balanced and adaptable cable-road layouts while allowing economic and environmental considerations to be incorporated into the design phase.

## Introduction

Cable yarding remains a productive system for timber extraction in steep terrain. Current European silvicultural strategies, coupled with environmental considerations, lead to smaller harvest areas and lower extraction volumes in recent years, shifting from clear-cutting to individual tree extraction. Notably, the time required for yarder installation, particularly in relation to the extraction time, has seen a significant increase, consequently raising the overall extraction costs (Schweier et al. 2020).

The digitalization of the cable-road planning process promises to lower installation and extraction costs by enabling a global optimization of the cable-road layouts. Recent GIS-based layout planning methods, however, often fall short for real-world applications, primarily due to the inadequate resolution of most remote sensing data, which typically relies on vegetation-density estimations with a spatial resolution of around 10 m for most bands (Acito et al. 2022). The resulting inaccuracies make the generated cable-road layouts less realistic and less suited for practical applications, as the placement of the cable roads only relies on estimation of tree densities instead of a concrete feasibility computation for each cable-road.

To overcome these constraints, we employed personal laser scanning (PLS) technology to generate high-resolution tree maps (Gollob et al. 2024). This method allows for the precise determination of individual tree locations, trunk attributes, diameters, and heights. By integrating these detailed tree maps with comprehensive digital elevation models in our previous work, we were able to compute precise cable-road representations. These cable roads include strategically placed intermediate supports and anchor points, ensuring cost-effectiveness and feasibility (Retzlaff et al. 2024).

In this paper, we extend our research by optimizing the selection of computed cable-road representations to design cost-efficient cable-road layouts. Initially, we quantify the costs and impacts associated with each cable-road, drawing on established productivity models outlined in previous studies (Ghaffariyan et al. 2009; Stampfer et al. 2013). We then create an optimized cable-road layout by selecting a combination of cable roads that efficiently cover the entire forest while minimizing multiple objectives (cost, residual stand damage, lateral yarding workload). To accomplish this, we employ a multi-objective approach, enabling us to balance the trade-offs between objectives and determine the most favorable overall outcome.

We compare a single-objective approach, a multi-objective optimization based on the epsilon-constraint method (AUGMECON) and a genetic algorithm (NSGA-II) with a layout created by an expert in forestry to show how these approaches can help create cost optimal layouts as well as allowing informed decision-making on the trade-offs between different objectives. This work contributes to the ongoing digital transformation in the field of smart forestry and aims to show how we can balance economical, ecological and ergonomical factors (Holzinger et al. 2022). Our work draws on three major research areas. Firstly, LiDAR modeling to quickly create 3D models of a specific area. Secondly, the computation of possible cable roads while considering mechanical factors including rope tension and deflection, anchor-holding forces, and optimal support placement. Finally, linear optimization approaches for selecting an optimal subset of the computed possible cable roads, leading to cost-optimal coverage of an area.

### LiDAR

LiDAR technology has become a valuable tool in forestry, providing high-resolution data of the forest environment through the creation of detailed 3D point clouds (Newnham et al. 2015; Marrs and Ni-Meister 2019). By emitting laser pulses and recording their reflections, LiDAR enables precise information about forest terrain, tree location, height, and canopy structure (Dubayah and Drake 2000). Terrestrial LiDAR, which uses ground-based scanning systems, excels in capturing accurate tree-level data, making it ideal for fine-scale forest inventory and monitoring. In contrast, aerial LiDAR, deployed from airborne platforms, offers broader coverage of large forest areas but struggles to penetrate dense canopies, limiting information on individual tree attributes (Popescu and Wynne 2004). While LiDAR-based simulations have the potential to support planning and evaluation processes in forestry, data accuracy depends on factors such as instrument quality, point cloud density, and weather conditions (Hancock et al. 2014). Therefore, aerial LiDAR is generally considered inadequate for obtaining comprehensive data on individual trees, while terrestrial LiDAR provides more precise tree-level information (Liang et al. 2014).

### Cable-road computation

The computation of the mechanical properties of cable roads in forestry has received considerable attention in research and was often improved alongside the optimization of cable-road layouts. The commonly found linear approach to modeling cable structures used in our previous work (Retzlaff et al. 2024) is based on Pestal (1961), which is relatively easy and fast to compute, but also relies on simplified assumptions (Bont and Heinimann 2012). A notable approach that also incorporates nonlinear assumptions to design optimal intermediate support layouts is the “close-to-catenary” approach by Zweifel (1960).

This approach takes into account the dynamic behavior of cables, considering factors such as cable tension, sag, and anchor point selection to ensure the structural integrity and stability of the cable-road system. Bont and Heinimann (2012) as well as the QGIS-plugin SEILAPLAN implement the approach by Zweifel and have been employed to solve cable-road layout problems at a large scale (Bont et al. 2022). SEILAPLAN allows considering various cable-road properties, including cable span clearance, terrain conditions, and environmental impact, to optimize the placement of individual cable roads and determine the most cost-effective intermediate support locations. It is however limited to the manual placement of cable roads, as usually there is no accurate tree map available.

### Layout optimization

There has been significant research on automatically determining optimal cable-road layouts (Sessions 1992; Chung and Sessions 2003). The location-allocation problem, for example, which has also provided insights applicable to forestry, is a well-known problem in this regard and has been extensively researched since the 1960s in the field of operations research (Bont and Church 2018). In the last decades, various optimization methods were introduced to identify cost-minimal harvesting units and allocate equipment, but approximation algorithms were still necessary for larger practical problems (Dykstra and Riggs 1977; Chung et al. 2004; Epstein et al. 2006).

Later publications presented set-covering models for addressing large-scale cable-road layout challenges (Bont and Church 2018). However, the emergence of both ecological and cost pressures necessitated the consideration of multiple objectives for a balanced outcome for different stakeholders. The most straightforward method for balancing multiple objectives involves manually adjusting the weights of objectives in a unified objective function (also referred to as weighted summation). Determining weights for this method is complex and typically relies on a blend of expert insights and experimentation with various weights (Bont et al. 2019), which is why alternative strategies were developed to computationally identify optimal solutions.

One of the first multi-objective approaches for balancing environmental impact and cost-effectiveness in forestry was presented by Bont et al. (2019). Multi-objective optimization aims to find a set of solutions that optimize multiple conflicting objectives simultaneously. Rather than finding a single optimal solution as described above, it identifies a range of solutions that represent trade-offs between these objectives, known as the Pareto front. The aim is to provide decision-makers with a range of options so that they can choose the most appropriate solution based on their preferences and priorities. A major limitation to the approach by Bont et al. (2019) was the reliance on low-resolution aerial GIS data, resulting in potentially infeasible layouts. To overcome this, we proposed an approach for incorporating high-precision forest stand maps generated by personal laser scanning and simulating cable roads based on this information, which enhances the reliability and applicability of the cable-road layouts (Retzlaff et al. 2024).

Two important multi-objective optimization approaches in recent times are NSGA (Non-Dominated Sorting Genetic Algorithm) and Epsilon Constraints (EC). NSGA is an evolutionary algorithm that evaluates and compares solutions based on multiple objectives simultaneously using non-domination sorting. It aims to find a diverse set of solutions along the Pareto front, promoting convergence and diversity. In contrast, EC transforms the problem into a series of single-objective sub-problems by introducing epsilon constraints that define acceptable ranges for each objective. It iteratively optimizes each objective subject to its corresponding epsilon constraint, exploring the feasible region of the objective space.

AUGMECON2 was introduced by Mavrotas and Florios (2013) and is an improved version of the EC method. The EC approach offers specific advantages over weighting summation, particularly in cases involving discrete variables (Mixed Integer or Pure Integer problems). It helps to avoid weakly Pareto optimal solutions, leading to faster convergence by preventing redundant iterations, and has demonstrated its effectiveness in various applications (Sedighizadeh et al. 2018).

While EC generally offer a straightforward approach for optimization, non-linear objective functions or discontinuous variable space can cause them to not be efficient. In these cases, heuristic methods like genetic algorithms (GAs) can provide useful alternatives. GAs are based on the principles of natural selection and genetic evolution, generating and evaluating a population of potential solutions iteratively. By exploring a wide range of solutions and employing genetic operators like crossover and mutation, GAs can find optimal or near-optimal solutions in complex problem domains. Their flexibility and adaptability make them well-suited for addressing real-world optimization problems that classical methods struggle with (Verma et al. 2021).

NSGA-II is an enhanced variant of the non-dominated sorting genetic algorithm (NSGA) and addresses several limitations of its predecessor, including the absence of elitism, the need to define sharing parameters for diversity preservation, and high computational complexity (Yusoff et al. 2011). NSGA-II eliminates the requirement for sharing parameters by employing a crowding distance operator to maintain diversity. Solutions with larger crowding distances have fewer neighboring solutions nearby, leading to a more diverse set of solutions. NSGA-II is also more computationally efficient as its overall complexity remains within an upper bound of O(MN2), where M represents the number of objective functions, and N denotes the population size (Verma et al. 2021).

NSGA and EC provide different approaches for multi-objective optimization, with NSGA focusing on diversity and EC on defined constraints, as illustrated by Bont and Church (2018). Furthermore, GAs like NSGA-II offer computational efficiency, as shown by Verma et al. (2021), addressing complex challenges with nonlinear objectives.

The overall objective of this work is to bridge the gap between realistic one-off cable-road planning based on individual trajectories and area-wide layout optimization relying on GIS data. This paper leverages the detailed cable-road representation from our previous publication (Retzlaff et al. 2024) to optimize cable-road layouts. The underlying code of this project can be found on GitHub (Retzlaff 2023). We compare the outcomes of a combined-objective approach aimed at minimizing the cost as well as residual stand damage and lateral yarding workload of a cable-road layout with an EC multi-objective optimization process with those generated using the NSGA-II (Non-Dominated Sorting Genetic Algorithm) optimization approach. We evaluate our results against an expert-generated layout to identify areas where our approach can be enhanced and quantify the extent of improvement it offers compared to expert knowledge.

## Materials and methods

The following section describes the underlying data of our optimization, methods used for quantifying the cable-road costs, as well as the approaches used for computing the optimization and details their implementation.

### Underlying data

The data acquisition and processing involved scanning a 1.62 ha forest stand using a GeoSLAM ZEB Horizon personal laser scanner, generating 3D point clouds using a SLAM algorithm, and extracting terrain and tree parameters using automatic routines developed with the R programming language. The scanning process requires around 40 min per hectare while also depending on factors like overall terrain difficulty and steepness.

The obtained parameters, including tree coordinates, height, volume, diameter at breast height (DBH), taper curves, and a digital terrain model, serve as the basis for optimizing the cable roads. The simulation of cable-road feasibility and parameters incorporates established computational methods in forestry, as described in Bont (2012),Gollob (2022) and Stampfer (2000). A comprehensive overview of rope deflection computation methods and required parameters is provided by Bont (2012), while additional details on anchoring, rope tensions, and cable-road loads are derived from discussions with an expert in forest technology (Gollob 2022). Geometric approaches for determining cable-road loads and supports are based on Stampfer (2000).

The expert-layout was generated in an informal context by the expert relying on a map of the possible cable roads, trees and knowledge about the corresponding forest area (Gollob 2022). The expert had 15 years of experience with cable yarding, owned a cable yarder, and had published one peer-reviewed manuscript in the field of cable yarding.

### Parametrizing cable-road costs and impacts

We optimize our cable-road layout based on three major factors (cost, residual stand damage, lateral yarding workload) to show how multi-objective optimization can help balance the inherent trade-offs between multiple objectives. The objective of residual stand damage helps to gauge the environmental impact a given layout has, while the lateral yarding workload objective helps to assess how much excess ergonomical workload is placed on the forestry worker. In the following paragraphs, we describe the motivation for choosing the different objectives. The approaches for the computation of the various objectives and their implementation can be found in Section “Optimization and Constraints.”

The first factor we consider is total harvesting cost. The drive toward smaller harvest areas and lower extraction volumes for ecological considerations has led to higher extraction costs (Schweier et al. 2020), which creates a major hurdle for the wider adoption of cable yarding systems. Therefore, we establish the primary goal of our optimization process as the reduction of total extraction expenses. We use the productivity models developed by Ghaffariyan et al. (2009) as well as the estimation of set-up and take-down time for cable roads by Stampfer et al. (2013) to estimate the costs associated with erecting a given cable-road as well as the cost efficiency (i.e. cost of m^3^ wood) for harvesting the nearby trees. We used the productivity models of Ghaffariyan et al. (2009) to calculate harvesting costs, which were created for motor manual felling and topping of the trees and delimbing and bucking at the landing.

As a secondary objective for the multi-objective optimization, we quantify environmental impacts to the forest stand by computing the residual stand damage (also referred to as stand damage) based on the lateral yarding distance beyond an established threshold. As the lateral yarding distance increases, so does the likelihood of damage to the remaining stand (Stampfer et al. 2003). This damage not only compromises the stand’s stability and vitality but also serves as an entry point for pathogens, which can lead to a decline in wood quality. The stability and vitality of a stand are key indicators for sustainable forest management (Kühmaier and Stampfer 2012). By measuring the lateral yarding distance, we can therefore gauge the potential residual stand damage and the subsequent reduction in tree value. The use of residual stand damage to gauge ecological impacts aligns with the growing emphasis on sustainable forestry practices, which are crucial for preserving natural habitats, preventing soil erosion and maintaining biodiversity, while also enhancing the public perception of the forestry industry (Rametsteiner et al. 2011).

As a tertiary objective, we quantify the ergonomical strain placed on workers with the lateral yarding workload (also referred to as yarding workload) they experience with increasing lateral yarding distance. During the lateral pull phase, the choker setter has to pull the steel cable and attach the choke to the felled trees, which becomes more straining with higher distances (Berendt et al. 2020). The increased strain is not just a matter of worker comfort but also has significant safety implications, as higher strain levels have been associated with increased accident rates in the forestry sector (Hoffmann et al. 2016).

The following paragraphs describe how the costs of all viable cable roads in the area are determined, which creates the basis for creating an optimal layout by selecting the cable roads with the lowest costs and best tree coverage. Equation 1 denotes the time per cycle required to fell and process a tree as per Ghaffariyan et al. (2009). The yarding distance in meters is the distance over which logs are transported. Each meter of yarding distance from the carriage to the tower yarder contributes 0.007 units to the minimum time per cycle. The lateral yarding distance in m is the horizontal distance from the yarding line to the tree. Each meter of lateral yarding distance from the carriage to the tree adds 0.043 units to the minimum time per cycle. The tree volume in cubic meters is the volume of the tree being harvested. The contribution of tree volume to the minimum time per cycle is inversely proportional to the cube root of the volume, with a coefficient of 1.307. The harvest intensity in percent is the percentage of trees being harvested from the overall stand. Each percentage point of harvest intensity adds 0.029 units to the minimum time per cycle. The slope in percent is the steepness of the terrain. Note that conventionally, cycle time decreases with higher harvesting intensity, due to reduced concern for damaging residual trees while harvesting. However, this coefficient indicates the opposite, ie. that higher harvest intensity leads to increased cycle time. Despite this unexpected relation, we found that the model by Ghaffaryian et al. (2009) provides the best overall predictions for cycle time. We hypothesize that the harvest intensity coefficient is either too weak to significantly impact the model’s predictive accuracy or may capture other unobserved interactions. Each percentage point of the slope contributes 0.038 units to the minimum time per cycle. Equation 1:

(1) $$\min/\mathrm{cycle}=0.007\times \mathrm{Yarding}\;\;\mathrm{distance}\left(m\right)+0.038\times \mathrm{Slope}+0.043\times \mathrm{Lateral}\;\;\mathrm{yarding}\;\;\mathrm{distance}\left(m\right)+1.307\times \mathrm{Tree}\;\;\mathrm{volum}e^{-0.3}\left(m\right)+0.029\times \mathrm{Harvest}\;\;\mathrm{intensity}\left(\%\right)+0.043\times \mathrm{Lateral}\;\;\mathrm{yarding}\;\;\mathrm{distance}\left(m\right)+0.029\times \mathrm{Harvest}\;\;\mathrm{intensity}\left(\%\right)+0.038\times \mathrm{Slope}$$

In keeping with the established practice of assigning the trees within a 15-m lateral distance to the cable-road (Ghaffariyan et al. 2009), we adopted a strategy that imposes a penalty on the cost efficiency of tree assignment to the cable-road (refer to Equation 2). To ensure that we do not overly constrain the optimization by ignoring trees slightly over 15 m, we penalize rather than constrain the tree assignment, allowing more flexibility in the cable-road layout planning. Consequently, for trees located more than 15 m from the cable-road, the excess distance is added as a penalty to the harvesting cost. Equation 2 defines this computation with *d* as the distance from tree to the cable-road and *pc* as the resulting penalty to cost, which makes trees farther from the cable-road comparatively more expensive to assign. This approach allows for the assignment of these trees if their location offers benefits that outweigh the additional felling costs and ensures a balance between cost efficiency and operational flexibility. Equation 2:

(2) $$\mathrm{pc}=\mathrm{pcif}\;\;d\leq 15\;\;\mathrm{pc}+\left(d-15\right)*2\mathrm{ifd}\;\;>\;\;15$$

The cost of set-up and take-down time of a cable-road is determined as per Stampfer et al. (2013) with the sum of Equations 3 and 4. The set-up time, measured in hours, is calculated as the exponential of a sum of factors. These factors include the cable-road length (in meters) multiplied by 0.00229, the intermediate support height (in meters) multiplied by 0.03, the corridor type (1 for first, 0 for subsequent installations to the same tower) multiplied by 0.256, the extraction direction (1 for uphill, 0 for downhill) subtracted by 0.65, the yarder size (in meters) multiplied by 0.11, and the product of the extraction direction and yarder size multiplied by 0.491.

Similarly, the take-down time, also measured in hours, is calculated as the exponential of a sum of factors. These factors include the corridor length (in meters) multiplied by 0.00233, the extraction direction subtracted by 0.31, the number of intermediate supports subtracted by 0.31, and the yarder size multiplied by 0.33. All these factors are summed and added to a constant of 0.96. These equations model the set-up and take-down times as exponential functions of these factors, each multiplied by its respective coefficient.

Note that the computed times are simplified models and actual times may vary based on other factors not included in these models and different environments, as different tree species, machinery, terrain and worker qualification can lead to vastly different set-up and take-down times.

Equation 3:

(3) $$\mathrm{Set}-\mathrm{up}\;\;\mathrm{time}(\mathrm{hrs})=e^{\mathrm{factors}}\mathrm{factors}=1.42+0.00229\times \mathrm{corridor}\;\;\mathrm{length}(m)+0.03\times \mathrm{int}.\mathrm{support}\;\;\mathrm{height}(m)+0.256\times \mathrm{corridor}\;\;\mathrm{type}-0.65\times \;\;\mathrm{extraction}\;\;\mathrm{direction}+0.11\times \mathrm{yarder}\;\;\mathrm{size}+0.491\times \mathrm{extraction}\;\;\mathrm{direction}\times \mathrm{yarder}\;\;\mathrm{size}$$

Equation 4:

(4) $$\mathrm{Take}-\mathrm{down}\;\;\mathrm{time}\left(\mathrm{hrs}\right)=e^{\mathrm{factors}}\;\;\mathrm{factors}=0.96+0.00233\times \mathrm{corridor}\;\;\mathrm{length}-0.31\times \mathrm{extraction}\;\;\mathrm{direction}-0.31\times \mathrm{int}\;\;\mathrm{support}+0.33\times \mathrm{yarder}\;\;\mathrm{size}$$

Both the productivity time per cycle and the set-up and take-down time for the cable roads are multiplied with the cost per man-hour, which is set to $44 USD ($60 USD adjusted for inflation in 2024) as per Stampfer et al. (2013).

We support both clear-cut and selective cutting as harvesting approaches. We implemented the A-value approach to select trees to fell based on the distance of trees clustered around a central Z-tree. The A-value is based primarily on two key factors: the height-to-diameter ratio (H/D) of the Z-tree and the diameter and proximity of neighboring trees. Johann (1982) formulated the A-value based on the following considerations: The stature of a Z-tree directly influences its spatial requirements within the stand. A neighboring tree assumes a competitive role (K) by infringing upon the space occupied by the Z-trunk (Z). When the height (H) of the competitor corresponds to that of the Z-tree, signifying an equivalent social position, the competitive pressure escalates as the tree height increases and the distance (E) between them decreases (H/E). Conversely, a tree of lesser stature or social standing exerts relatively lower competitive pressure on the Z-trunk than a more robust tree. This observation can be expressed as the ratio of the competitor’s diameter (d) to that of the Z-trunk (D). Given those considerations, a competitor tree in a cluster is removed when its distance from the Z-tree falls below a specified critical distance (GD), provided a fixed A-value criterion is met Equation 5:

(5) $$\mathrm{GD}<H/\left(A*d/D\right)$$

We utilize Equation 5 to automatically select all trees which fall below the threshold for cutting, allowing to easily adapt our approach to selective cutting.

### Optimization approaches

As described previously, we employ three different optimization approaches to create an optimal layout of cable roads.

*Single-Objective Optimization (SOO)*: We apply a single-objective optimization approach to cable-road layout design with the weighted addition of individual objectives. Leveraging the Python packages spopt (Feng et al. 2022) and pulp (Mitchell 2011), we formulate the optimization problem, modeling cable-road placement as a linear programming task. We use the Coin-OR solver to determine an optimal cable-road layout which offers the lowest installation expenses, balanced with the secondary objectives. Finding weights for this approach is challenging and often based on a mixture of expert knowledge and experimenting with different weights (Bont et al. 2019). This process can be tedious and fails to discover all optimal solutions, which motivates the move toward true multi-objective approaches. We employ SOO in this work to have a comparative baseline for more advanced approaches, as well as to compute Nadir points, i.e. the worst possible objective values found in the Pareto front, for the individual objectives, as required by AUGMECON2 (Mavrotas and Florios 2013). As reference point for the other approach, we implement a combined-objective approach (see Equation 6) which minimizes the equally weighted sum of the individual objectives (Equations 9, 10, 11). The equation minimizes the sum of the cost objective, the residual stand damage objective and the lateral yarding workload objective. This simple addition of different factors is the established way to combine objectives and is usually fine-tuned by adjusting the individual objective weights in an iterative way and with the help of expert knowledge (Yang 2014) Equation 6:

(6) Min∑1nCostobjective+Ecologicalobjective+Ergonomicalobjective

*AUGMECON2*: Secondly, we apply a slightly modified version of the AUGMECON2 multi-objective optimization algorithm to the cost optimization problem. AUGMECON is part of the family of epsilon-constraint approaches and formulates secondary objectives as constraints, which allows considering secondary objectives alongside the primary cost objective (Mavrotas and Florios 2013). AUGMECON enables a systematic evaluation of cable-road layouts with different objective weightings and provides a balance on the spectrum of implementation effort and performance between manually weighting objectives as in SOO and more elaborate Genetic Algorithms like NSGA-II. We chose to not adapt the AUGMECON2 version of formulating the optimization objective over the original AUGMECON implementation, as the secondary objectives are equally important in our application. We furthermore modify the objective function to minimize instead of maximizing the objective. We selected a step-size of four for the grid-point search, which results in 16 evaluations at most and provides a balance between fine-grained results and computation time. See Equation 7 for the objective function and Equation 8 for the constraints. In Equation 7, we minimize the main function f*1(x)*, which corresponds to the cost function in our case, while also minimizing the product of the surplus variable *S* (how much the objective is better, i.e. lower, than the expected value) divided by the range *r* (the globally minimized value of this objective) of all other objectives. This is subject to a lower limit (Equation 8), where the objective value of the secondary functions minus the surplus variable has to be equal to the expected value *e* (which is determined by a grid of target objective values from least to most desirable objective value). Equations 7 and 8:

(7) $$\mathrm{Min}\;\;f_{1}\left(x\right)+\varepsilon \cdot \left(\frac{S_{2}}{r_{2}}\cdot \frac{S_{3}}{r_{3}}+\ldots \cdot \frac{S_{p}}{r_{p}}\right)$$

(8) $$s.t.f_{2}\left(x\right)-S2=e2\ldots f_{p}\left(x\right)-S_{p}=e_{p}$$

*NSGA-II*: To complement the previous approaches, we also implement the NSGA-II (Non-dominated Sorting Genetic Algorithm II) method for cable-road layout design. Unlike the AUGMECON approach, NSGA-II operates through a genetic algorithm paradigm, introducing variability and exploration in the optimization process. We frame the optimization as a binary decision problem, where each cable-road can be either open or closed and the algorithm starts with only one randomly selected cable road. For selecting cable roads within the genetic algorithm, we implement the simulated annealing optimization approach. We first define a temperature *t* that decreases with each iteration. The difference between the objective function values before and after a new solution is proposed and is calculated as *diff_objectives*. The metropolis criterion, a probability that depends on this difference and the current temperature, is then calculated. If the new solution improves the objective function, it is accepted. If not, it is still accepted with a probability given by the metropolis criterion. This allows the algorithm to avoid local minima by occasionally accepting worse solutions. If the new solution is not accepted according to these conditions, it is rejected. This process is repeated until a stopping criterion is met, a sufficiently low temperature or, in our case, more than 100 iterations. This probabilistic optimization scheme is used in various applications and is known for fast convergence and its relative simplicity (Suman and Kumar 2006). Code Listing 1:

*t = temp/iteration*

*diff_objectives = objective_before – objective_after metropolis_criterion = exp^(-diff_objectives/t)*

*if objective_after < objective_before:*

*accept_new_solution()*

*elif rand(0,1) < metropolis_criterion*:

*accept_new_solution()*

*else:*

*reject_new_solution()*

We run the NSGA-II algorithm with 10 different solutions for 20 generations, which in our case resulted in optimal results, as indicated by decreasing changes in the objectives around the 12th generation and converging at the final generations.

NSGA-II holds the potential advantage over AUGMECON with its ability to explore a wider solution space even in non-convex spaces. The random mutation strategy in NSGA-II introduces an element of randomness that can help escape local optima and discover a diverse set of solutions. This contrasts with AUGMECON’s more linear and systematic approach.

### Optimization and constraints

In the following section, we describe how the objectives and constraints described in the previous sections were implemented to solve the problem of optimal cable-road layouts. The main objective is defined in Equation 9 and is also referred to as cost objective. It minimizes the overall cost for all cable roads selected (*f* multiplied by a binary *select* variable), as well as the cost of retrieving the trees assigned to each cable-road (*c* multiplied by the corresponding binary *assign* variables) Equation 9:

(9) $$\mathrm{Min}\;\;Z=\sum j\in J\;f_{j}\cdot \mathrm{selec}t_{j}+\sum j\in J\;\sum i\in I\;c_{i,j}\cdot \mathrm{assig}n_{i,j}$$

The second objective (see Equation 10), also referred to as the residual stand damage objective, minimizes environmental impact by considering yarding distances above 10 m as ecologically unfavorable. The established average lateral yarding distance in thinning operations is 10 m (resulting in an 20 m rope alley spacing), because damage to the remaining stand increases disproportionately at higher lateral draw distances (Stampfer et al. 2003; FHP Kooperationsplattform Forst - Holz - Papier 2019). We classify these damages as exponentially detrimental according to (Stampfer et al. 2003)), and, therefore, penalize lateral yarding distances over the established threshold with the square of the excess distance. Equation 10 minimizes the sum of yarding distances by the *select* variables, i.e. the selected trees, as well as their corresponding lateral distances to their cable road if larger than 10 m, squared Equation 10:

(10) $$\mathrm{Min}\sum j\in J\;\mathrm{selec}t_{j}\cdot {\left(l\mathrm{ateraldistance}>10m_{j}\right)}^{2}$$

The third objective (see Equation 11), also referred to as the lateral yarding workload objective, minimizes the physical workload of the worker by penalizing lateral yarding distances over 15 m, oriented at the lateral yarding distance considerations for worker productivity by Ghaffariyan et al. (2009). During the yarding operation, the choker setter has to pull a steel cable and attach the choker to the felled trees, which becomes increasingly strenuous as the distance increases and has been associated with higher accident rates in the forestry sector (Hoffmann et al. 2016; Berendt et al. 2020). We choose to set this threshold similarly to the yarding cost estimation, acknowledging the direct connection between both operations and their linear increase with excess distance. Equation 11 minimizes the sum of lateral yarding distances for all trees which are enabled by the *select* variable multiplied by their corresponding lateral yarding distance, if it is larger than 15 m Equation 11:

(11) $$\mathrm{Min}\sum j\in J\;f_{j}\cdot \mathrm{selec}t_{j}\cdot \mathrm{lateral}\;\;\mathrm{distance}>15m_{j}$$

The optimization is constrained by Equation 12 to ensure that all trees are assigned to exactly one cable-road, and by Equation 13 to ensure that each cable-road which has a tree assigned must be selected. Equation 12 defines that the sum of each *assign* variable, where the *assign* variables are a matrix of binary variables for each tree to each cable corridor, has to be exactly 1, which forces the algorithm to assign each tree to exactly one cable-road Equation 12:

(12) $$\sum j\in J\;\mathrm{assig}n_{i,j}=1\mathrm{\forall i}\in I$$

Equation 13 requires that if tree *I* is assigned to a cable-road *j*, the *select* variable must be 1, i.e. the cable-road must be built Equation 13:

(13) $$\mathrm{assig}n_{i,j}\leq \mathrm{selec}t_{j}\forall i\in I\forall j\in J$$

In the process of the AUGMECON implementation, we are iteratively solving the optimization problem in Equations 14, with SS being the slack of the stand damage objective, RS being the range of the stand damage objective, DS the yarding workload objective, R_S_ the yarding workload range and e the corresponding expected value in the linear grid from maximal to minimal objective value. The secondary and tertiary objectives are reframed as weighted constraints (see Equation 15). Deviating from the implementation by Mavrotas and Florios (2013), we convert the problem to a minimization problem as opposed to a maximization in the original AUGMECON approach and chose to keep an equal weighting of the secondary objectives as opposed to AUGMECON2 Equations 14 and 15:

(14) $$\mathrm{Min}f_{c}\left(x\right)+\varepsilon \cdot \left(\frac{S_{S}}{R_{S}}\cdot \frac{S_{D}}{R_{D}}\right)$$

$$s.t.f_{s}w\left(x\right)-S_{S}=e_{D}$$

…S_S_R_S_D_S_R_S_e

(15) $$f_{d}h\left(x\right)-S_{D}=e_{D}$$

To iteratively constrain the secondary objectives, AUGMECON needs to define minimum- and maximum values for the secondary objectives. For finding the range of these parameters with a single-objective optimization, we limit the maximum number of cable roads to a given number (five in our case) to prevent the algorithm from simply selecting all cable roads when only minimizing the secondary objectives (see Equations 11 and 12). The underlying code and the results of the planning process are publicly available in the GitHub repository (Retzlaff 2023).

## Results

**Figure 1.**
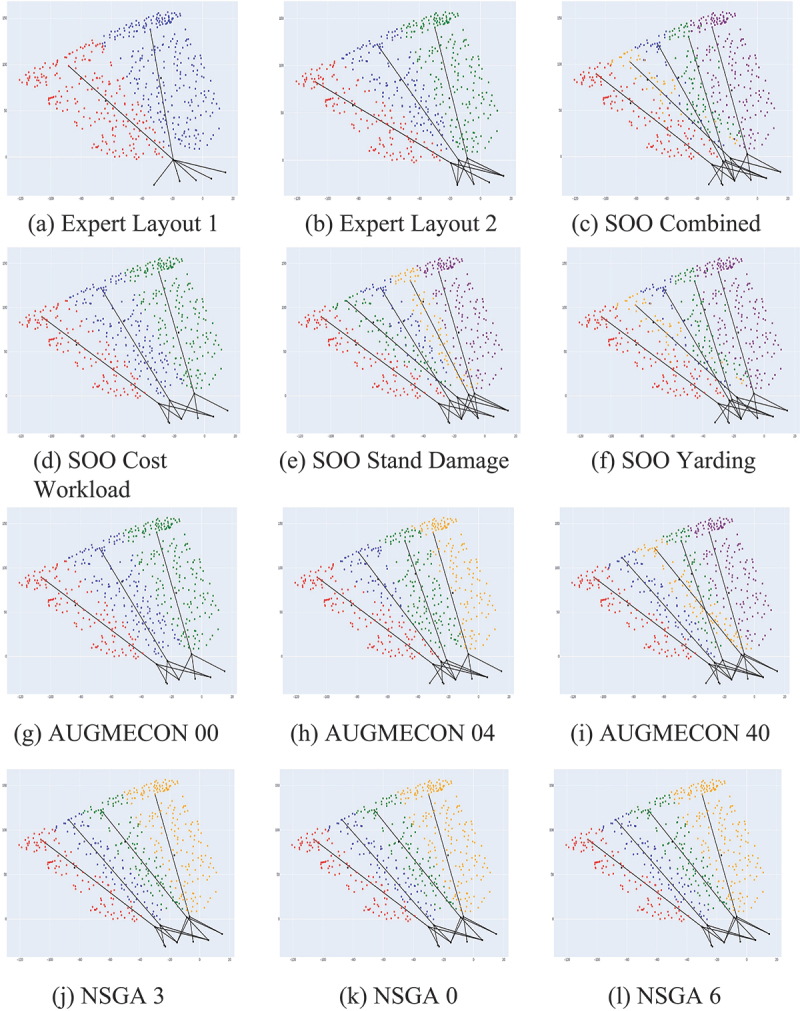
Comparison grid of cable-road layouts per technique. The dots represent trees, and their color the cable-road they are assigned to. The long black lines denote activated cable roads, and the three smaller lines their anchoring configuration.

The results of the different optimizations are summarized in Table 1 to provide a comprehensive overview of the various cable-road optimization approaches employed in this study, including the single-objective optimization (SOO), AUGMECON2, NSGA-II, and the expert-designed layout. See Appendix Figure S1 for a visual comparison of these results and Figure 1 for how the corresponding layout looks like applied to our forest. Appendix Figure S2 shows a 3D view of the AUGMECON-00 layout plotted on the ground surface point cloud. Appendix Figures S3 to S14 show the corresponding large-scale version of the layout of each model. Since many similar solutions were generated, we show only three results per approach in Table 1, while Appendix Table S1 provides an extended version with all model results. Similar to the approach from Bont et al. (2019), we show the results as relative maximal impact by comparing the best case of each objective within the other scenarios. This has the benefit of removing the need for manually weighting the objectives as in the SOO approach, and instead focusing on the relative impact to the best and worst case for each objective.

**Table 1.** Comparison of the relative impact of each approach for the objective cost, residual stand damage and yarding workload. The numbers in percent show how much each objective is fulfilled in comparison to the best possible solution, focusing on only one objective. I.E. when expert layout 1 achieves a 63%, it has 37% higher costs than the 100% cost objective only solution.

Model name	Cost Objective	Stand Damage Objective	Yarding Workload Objective
SOO Cost Objective	100	57	60
SOO Stand Damage Objective	80	100	100
SOO Yarding Workload Objective	76	95	100
SSO Combined Objective	93	77	83
NSGA-II 5	93	78	82
NSGA-II 0	91	85	90
NSGA-II 6	93	79	78
Augmecon 00	100	57	60
Augmecon 04	88	88	96
Augmecon 40	85	99	100
Expert Layout 1	63	19	11
Expert Layout 2	69	47	39

For single-objective SOO layouts, four distinct approaches were considered: SOO Cost, focusing solely on cost optimization; SOO Stand Damage, emphasizing the minimization of ecologically unfavorable distances; and SOO Yarding Workload, prioritizing the minimization of ergonomically unfavorable yarding distances. The SOO Combined Objective is an equally weighted combined objective. In the following, we refer to the cost objective as per Equation 9, the residual stand damage objective as per Equation 10 and lateral yarding workload objective as per Equation 11. Then, we show three selected results by NSGA-II and from the AUGMECON approach (see B for all resulting layouts). Finally, we evaluate two cable-road layouts generated by a forestry expert with the help of an interactive tool.

Our results are presented in terms of relative negative impact, indicating the percentage difference from the best achievable value as per the single-objective approach, which always achieves the overall minimum objective value. This means that, for example, an 88% solution for the cost objective requires 12% more costs than the most cost-effective solution possible. Solution “SOO Stand Damage Objective” optimizes only for ecological factors and offers a well-balanced solution with only a 20% penalty from optimal costs. Conversely, “SOO Yarding Workload Objective” optimizes for ergonomic factors in isolation and yields suboptimal solutions, as the optimization process terminates prematurely upon reaching the minimum for lateral yarding workload.

The manually combined objective approach (“SOO Combined Objective”) results in a 7% reduction in costs while simultaneously improving the stand damage and yarding workload objectives by 20% and 23%, respectively, striking a favorable trade-off.

AUGMECON’s stepwise approach offers the flexibility to trade off objectives systematically, a valuable feature in optimization. We can see that the stand damage and yarding workload objectives are becoming improved in higher iterations, trading the cost objective. We evaluated five steps for AUGMECON, while a more fine-grained approach can be used to discover the true Pareto frontier. A solution like “Augmecon 40” for example shows how with a 1% drop of the stand damage objective, the cost objective can be improved by 5%. AUGMECON, while computationally expensive, helped to explore the entire decision space.

NSGA exhibits efficiency in terms of computational speed while also providing viable optimization results. For instance, the “NSGA-II 5” solution closely approximates the “SOO Combined Objective” solution, showing that it approximates global optima. Furthermore, the “NSGA-II 0” solution illustrates the feasible trade-offs between objectives. An example of this is how a minor increase in cost (a penalty of 2%) can lead to substantial improvements in the stand damage objective (by 7%) and the yarding workload objective (by 9%). This highlights the algorithm’s ability to balance multiple objectives effectively and come up with unique solutions.

The expert layout, which is visually selected based on a 2D and 3D model of the area, incurs a substantial profit sacrifice (up to 31%) and has much higher relative negative stand damage (47%) and yarding workload (39%) impact. One should note, however, that the overall layout closely resembles optimal solutions. Since the forestry expert has less of an overview of how costly the individual computed cable roads are, this shows that both the forestry expert and the optimization algorithm “agree” on what a good layout should broadly look like.

Table 2 gives an overview of the computation times for each optimization approach and how they scale with an increasing number of objects.

**Table 2.** Comparison of execution times. SOO and AUGMECON quickly get slower with increasing number of cable roads and trees. NSGA-II performs worse with less cable roads and trees, as it stalls with searching for improved configurations in this too small decision space but has a better performance for larger areas.

Model Name	100 Trees 10 Cable Roads	200 Trees 50 Cable Roads	500 Trees 70 Cable Roads
SOO	0.007s	2.3s	12.3s
NSGA-II	23.9s	12.7s	38.2s
AUGMECON	5.5s	30.2s	227.1s

For the full layout, the SOO approaches require 17 s in total, while the NSGA approaches, with 10 populations and 20 generations, take 29 s. In contrast, AUGMECON necessitates 2 min and 45 s. The computation time per solution in AUGMECON aligns with that of SOO, but the larger set of solutions accounts for the longer overall computation time. AUGMECON provides the strongest option for an exact computation of the Pareto frontier, while NSGA-II scales better in larger settings. It, however, also comes with a longer set-up time for smaller layouts, since it initially requires loading a lot of data. The expert needed a negligible time for deciding on their layouts in comparison with the computation time of the other approaches.

## Discussion

We found that NSGA-II is a valuable tool for optimizing the cable-road layout, as it exhibited a good performance and found near-optimal solutions in our cable-road layout optimization task. However, implementing NSGA-II was a demanding process, requiring a significant investment of effort and expertise, since mutation, repair and crossover operations must be manually defined. This is different from the conventional location allocation problem formulation (as implemented with our single-objective approach), which already has many comparable implementations available. We find that NSGA is particularly promising for larger-scale optimizations, which comes at the cost of development time and effort. Utilizing simulated annealing further opens up new cable-road combinations, making the approach less likely to get stuck in local minima. During the implementation process, we found several possible improvements of the NSGA implementation, which could further improve the performance and quality of results. Both mutation and crossover implementations could aim to directly minimize the distances of the cable roads to the trees instead of semi-randomly selecting new mutations, likely increasing the speed of convergence, but at the cost of discovering all possible solutions.

Generally, we show how a multi-objective approach can balance costs, residual stand damage and lateral yarding workload. We quantify residual stand damage with lateral yarding distance, and its association with residual stand damage. We, however, want to highlight that we ultimately want to measure the overall environmental impact to the forest stand, instead of just the residual stand damage, which would encompass various other factors besides stand damage and the associated vitality and health of the trees. Our focus on stand damage highlights the importance of stand health in our target audience for forestry as well as the ability to quantify it appropriately with lateral yarding distance. Further research could encompass other factors into the calculation of the environmental impact such as soil damage, impacts on ecological diversity, nutrient loss, erosion, detriments to water quality, etc. Similarly, we propose to move toward quantifying the overall ergonomical impact of a layout on forestry workers with factors such as worker heart rate above a healthy range (Arman et al. 2021) as well as accident rates (for example, accidents per million cubic meters of wood harvested). This, however, also creates the necessity for research in how to quantify these impacts depending on factors in the cable yarding process (amount of supports, height of the cable above ground, total length of cable corridors, etc.).

One factor that we highlight for environmental impact specifically is full vs. partial suspension of the felled tree since dragging the stem across the ground can significantly impact soil health (Spinelli et al. 2021). When considering the environmental impact caused by the stems being dragged across the ground, we, however, argue that it is not sufficient to only consider the deflection of the cable-road. The weight and height of the log to be moved, which are not known ahead of time, should also be taken into account. A simplified approach could be to compute a standard load (e.g. a log of certain DBH, length, and weight) and then compare the length of the section where it would have skidded across the forest floor. To quantify factors such as worker heart rate, it would be beneficial to conduct a study investigating its correlation with other, easily computable factors such as the size of the felled trees, the condition of the terrain (including aspects like steepness), and the yarding distance, which could in turn allow a better quantification of the ergonomic impact of forestry operations. For future work, we recommend conducting a literature review and assembling a multidisciplinary team to explore possible factors and ways to quantify them.

We furthermore note that both residual stand damage and lateral yarding workload are modeled based on lateral yarding distance, which leads to a potential correspondence between both factors. We argue that the difference in penalty thresholds as well as different growth patterns (exponentially for residual stand damage as per Stampfer et al (2003), linear for lateral yarding workload as per Berendt et al. (2020)) leads to a different weighting of the impacts but still recommend the inclusion of other suitable and quantifiable factors in further work.

A general limitation of our approach is that it relies heavily on the quality and availability of data, since the accuracy and reliability of the solutions we generate are directly influenced by the data used as input for the computation of the cable roads. Therefore, if the data is incomplete or of poor quality, it can negatively impact the effectiveness of our solutions. Furthermore, to apply our optimization process to other areas, terrestrial LiDAR data must be acquired for a given area, which requires both personnel with expertise with PLS-devices and costs for manual labor and equipment. Analyzing LiDAR data, which takes approximately 4 h of computational time for every 40 min of scanning per hectare, as well as affording a 30.000€ laser scanning device (as used by Gollob et al. 2024) presents a significant challenge for less technically involved users. Furthermore, the expertise and methodologies for interpreting LiDAR data are predominantly confined to specialized academic circles and a handful of commercial operators, which prevents the wider public from accessing LiDAR scanning across a range of practical applications. To overcome these obstacles, it is crucial to simplify access to LiDAR data processing techniques beyond mainly academical applications.

Another limitation relates to the comparison of our results against those of a forest expert. While within our optimization domain, we strive to achieve the best possible solution, it does not necessarily guarantee that our results will be superior in real-world scenarios. It is important to acknowledge that the expertise and experience of a forest expert can still provide valuable insights and considerations that may not be captured solely through optimization. Therefore, although we may have achieved optimal solutions within our domain, their real-world performance and superiority may still require further evaluation and validation.

Finally, the underlying assumptions for determining the cable-road costs could be further improved to generate more realistic results. The function for determining the set-up and take-down time by Stampfer et al. (2013) was adopted for large distances between cable-road and trees found in our model, while trees more than 15 m away are simply not considered in their model. The same applies to the cost-efficiency model by Ghaffariyan et al. (2009), which is not intended for larger distances as found in our model. We confirmed in our discussion with a forestry expert that those assumptions are still reasonable, but a more fine-grained model for determining the costs will greatly help with better results. In particular, given the great diversity of forests around the world and the associated changes in harvesting times, set-up times, residual stand damage, etc., we emphasize the need to adapt the underlying assumptions of our models to the given situation.

## Conclusions

Our research focused on the optimization of cable-road layouts using terrestrial LiDAR data. We conducted a comprehensive analysis of different multi-objective cable-road layout optimization approaches based on set-up and take-down times, productivity costs, residual stand damage and lateral yarding workload. We compared single-objective linear optimization with AUGMECON and NSGA-II and a layout set by a forestry expert. The results showed that both AUGMECON and NSGA produced commendable results, with NSGA standing out for its significantly faster performance, while the layout proposed by the forest expert imposed high penalties on all objectives, while broadly resembling the results of a combined-objective approach.

We find that the notable strength of our approach is its ability to highlight the trade-offs inherent in the optimization process, which provides valuable insights into the decision-making dynamics within the field of cable-road layout design, allowing, for example, small cost increases to be traded off against large reductions in residual stand damage or lateral yarding workload.

Looking ahead, we propose to develop interactive interfaces to provide in-depth explanations of the inner workings of our solutions. In addition, we aim to validate and test our approaches in real-world settings, bridging the gap between theory and practice. We hope that these results will also contribute to the wider adoption and impact of optimization approaches in the field of forestry and cable-road design.

## Supplementary Material

Supplemental Material
