# Supplementary material for: Multi-objective optimization of cable-road layouts in smart forestry
Source: Int J For Eng. 2024 Aug 11;35(3):444–55. doi: 10.1080/14942119.2024.2380229 (PMC11583030; doi:10.1080/14942119.2024.2380229)
Supplement: Supplemental Material [file TIFE_A_2380229_SM7764.docx]

# Appendix

## Tables

*Appendix Table 1: Comparison of all results*

| **Model name** | **Cost**  **Objective** | **Residual Stand Damage** | **Yarding Workload** |
| --- | --- | --- | --- |
| SOO Cost Objective | 100 | 57 | 60 |
| SOO Stand Damage Objective | 80 | 100 | 100 |
| SOO Yarding Workload Objective | 76 | 95 | 100 |
| SSO Combined Objective | 85 | 87 | 96 |
| NSGA2 5 | 93 | 78 | 82 |
| NSGA2 3 | 93 | 77 | 83 |
| NSGA2 0 | 91 | 85 | 90 |
| NSGA2 6 | 93 | 79 | 78 |
| Augmecon00 | 100 | 57 | 60 |
| Augmecon03 | 91 | 85 | 90 |
| Augmecon04 | 88 | 88 | 100 |
| Augmecon10 | 92 | 67 | 75 |
| Augmecon13 | 91 | 85 | 90 |
| Augmecon14 | 85 | 99 | 100 |
| Augmecon20 | 93 | 77 | 83 |
| Augmecon23 | 91 | 85 | 90 |
| Augmecon24 | 85 | 99 | 100 |
| Augmecon30 | 91 | 85 | 90 |
| Augmecon34 | 85 | 99 | 100 |
| Augmecon40 | 85 | 99 | 100 |
| Expert Layout 1 | 58 | 19 | 11 |
| Expert Layout 2 | 69 | 47 | 39 |

##
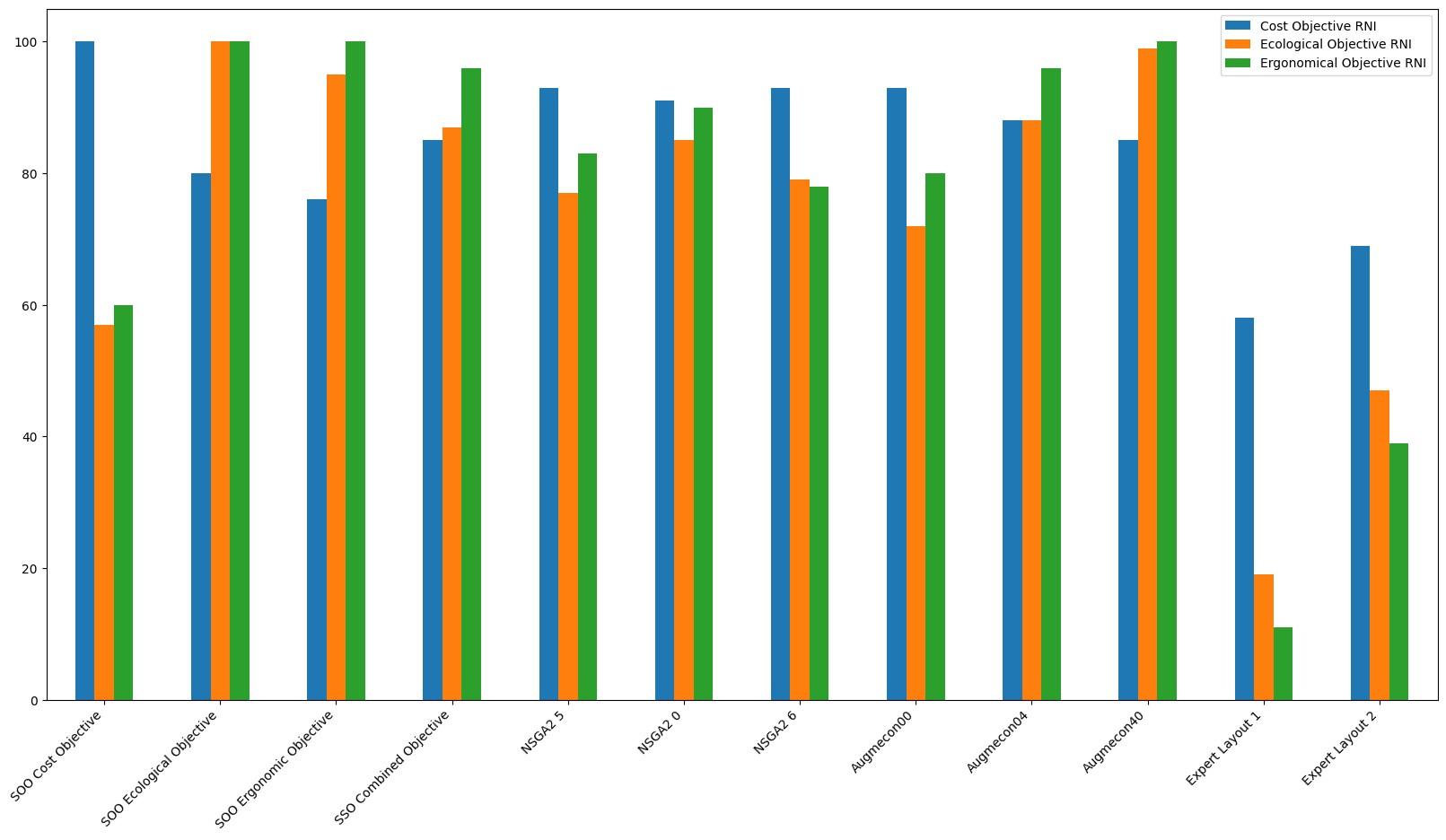
Images

Appendix Figure 1: Comparing the objective values of the respective layouts.


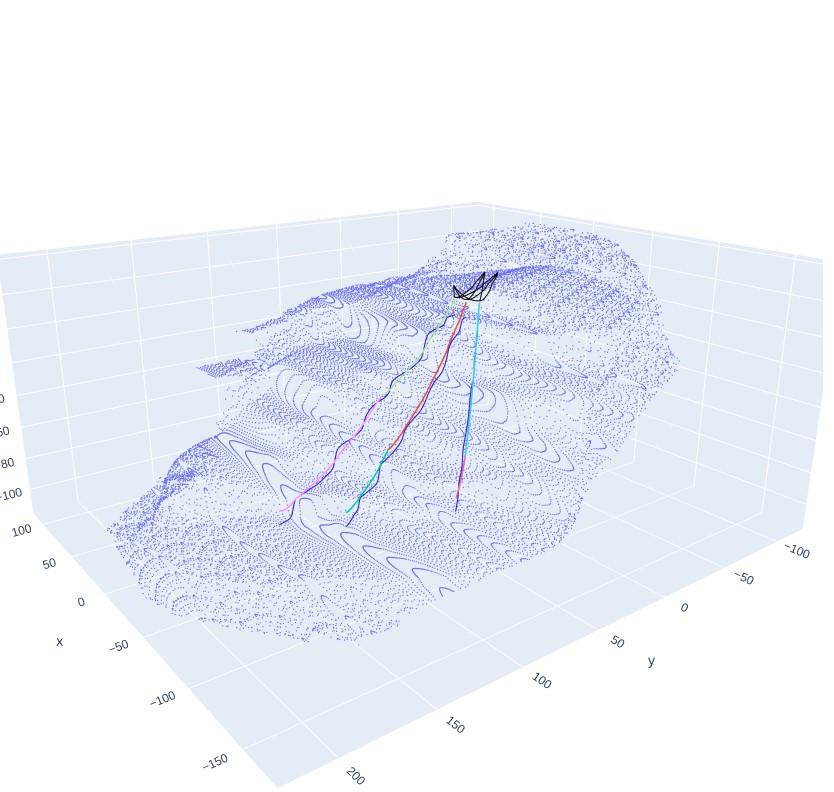


Appendix Figure 2: Plotting the AUGMECON-00 layout in 3D.


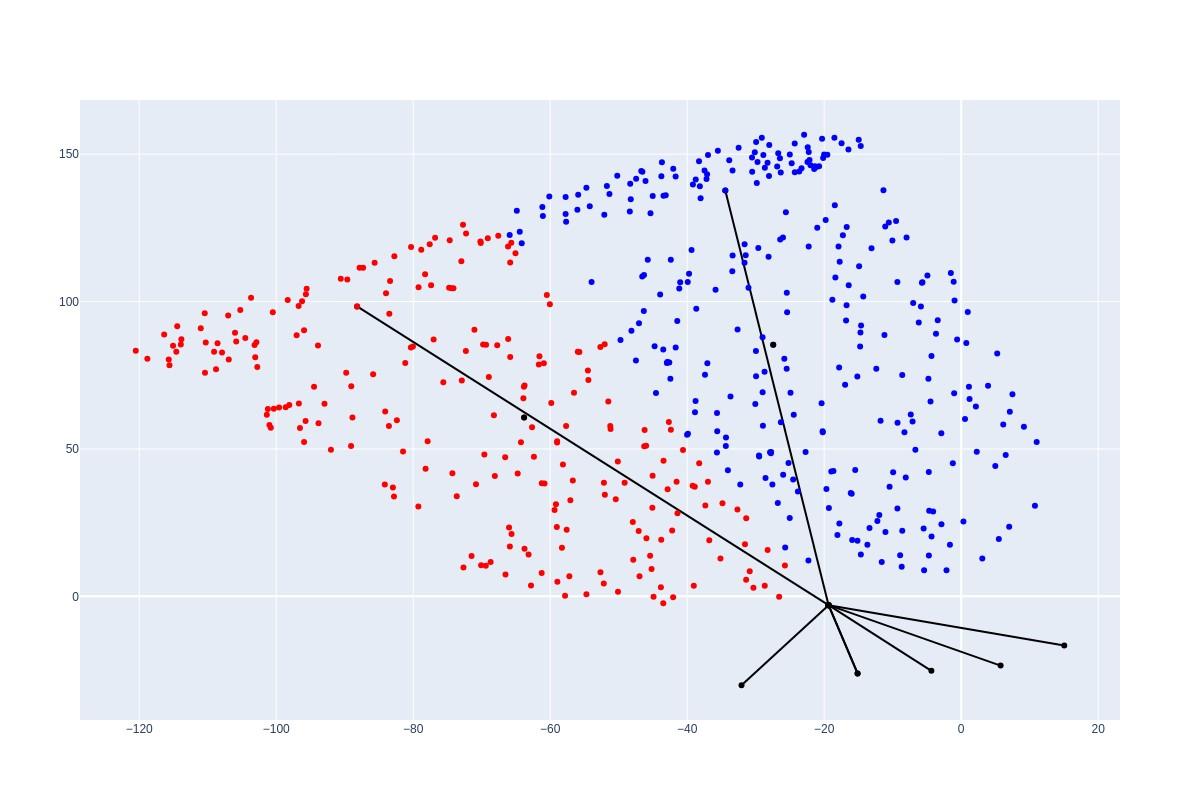


Appendix Figure 3: Expert Layout 1


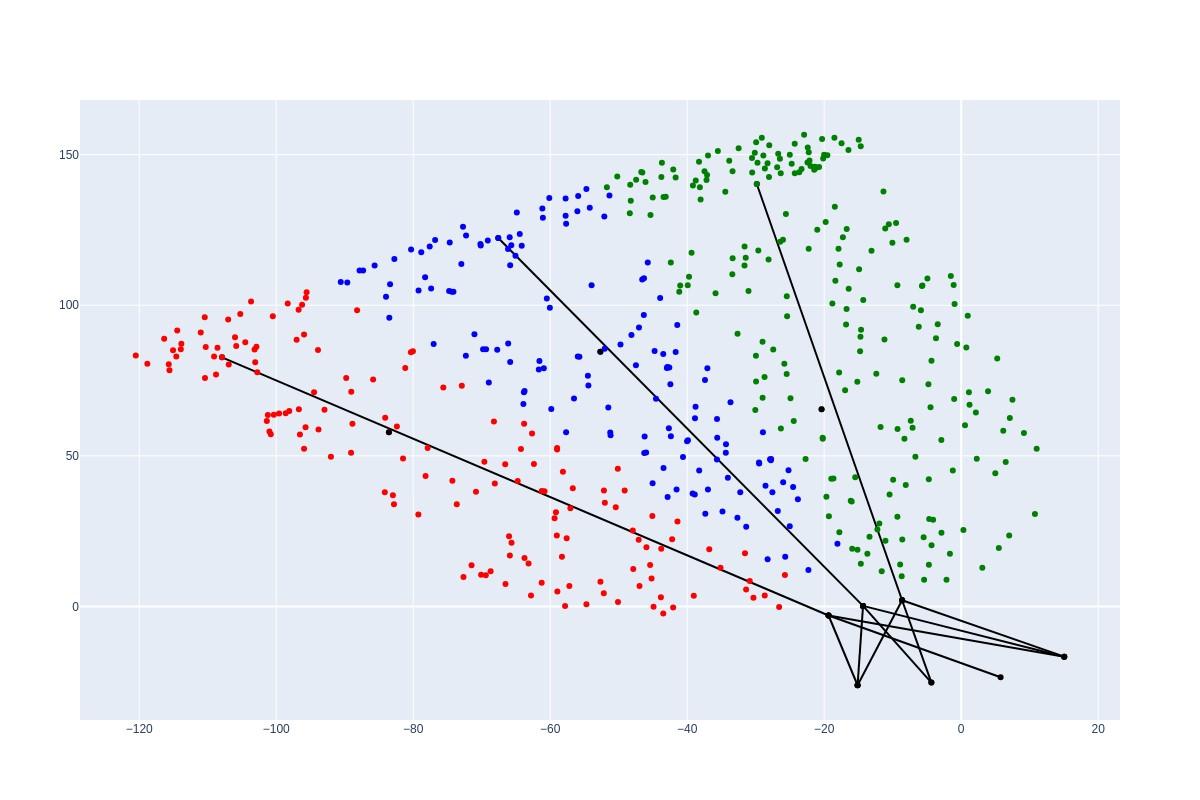


Appendix Figure 4: Expert Layout 2


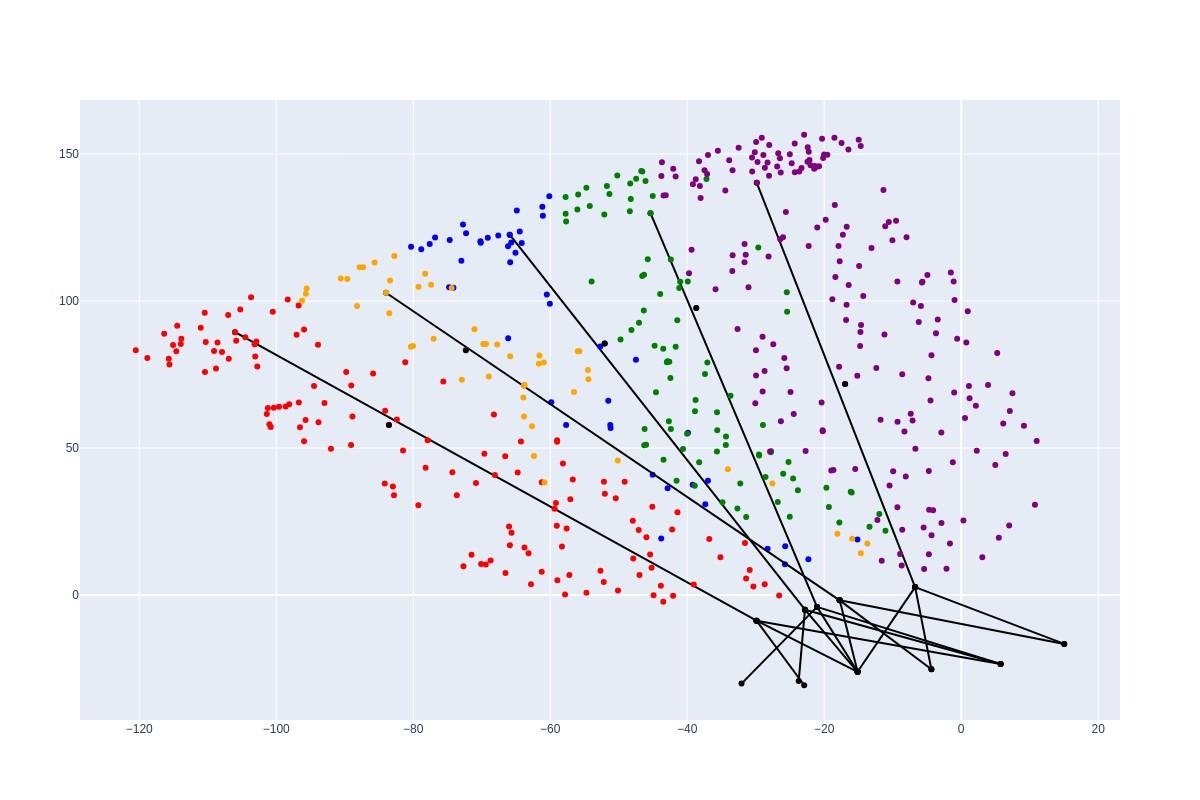


Appendix Figure 5: SOO Combined


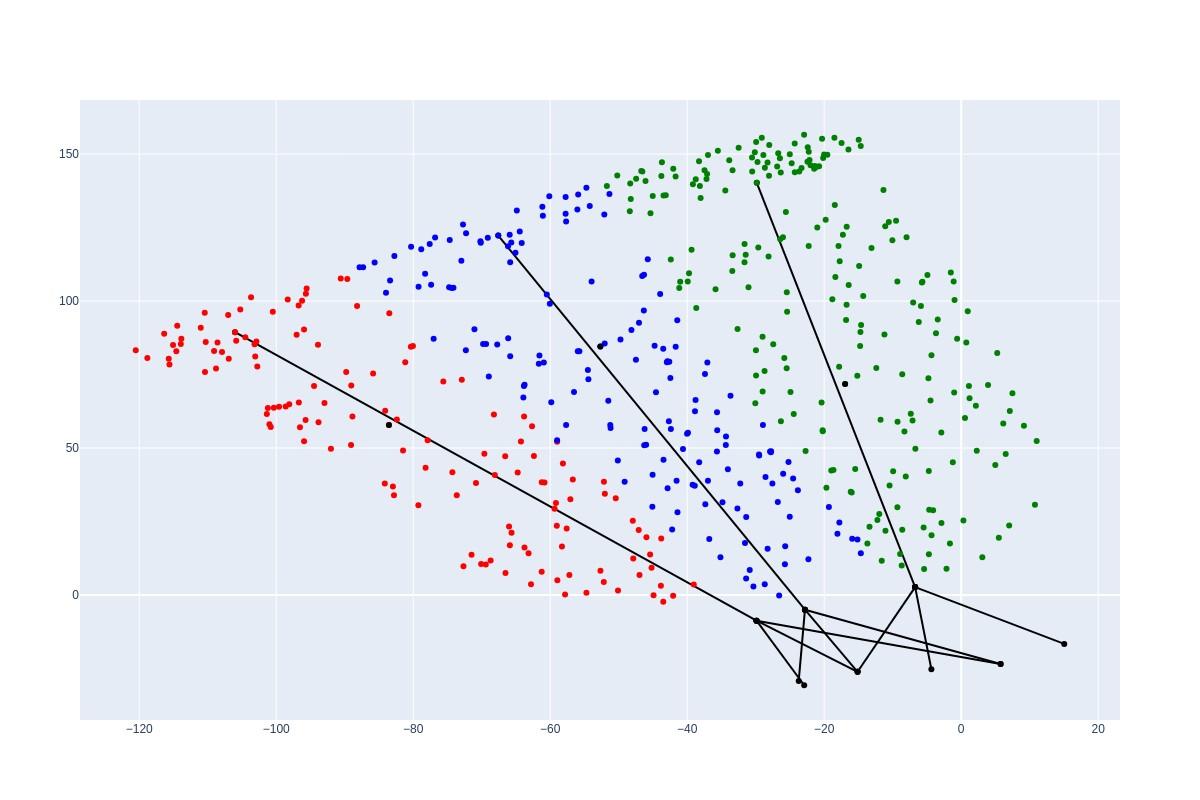


Appendix Figure 6: SOO Cost


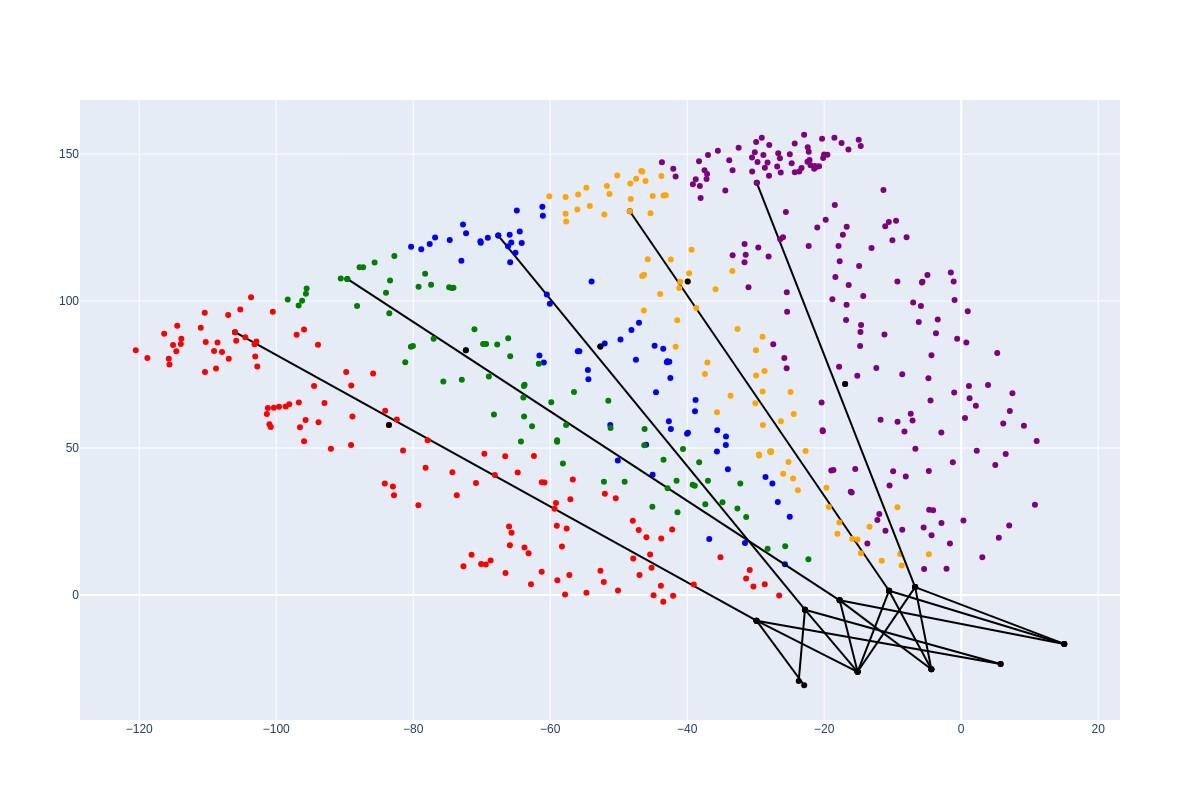


Appendix Figure 7: SOO Ecological


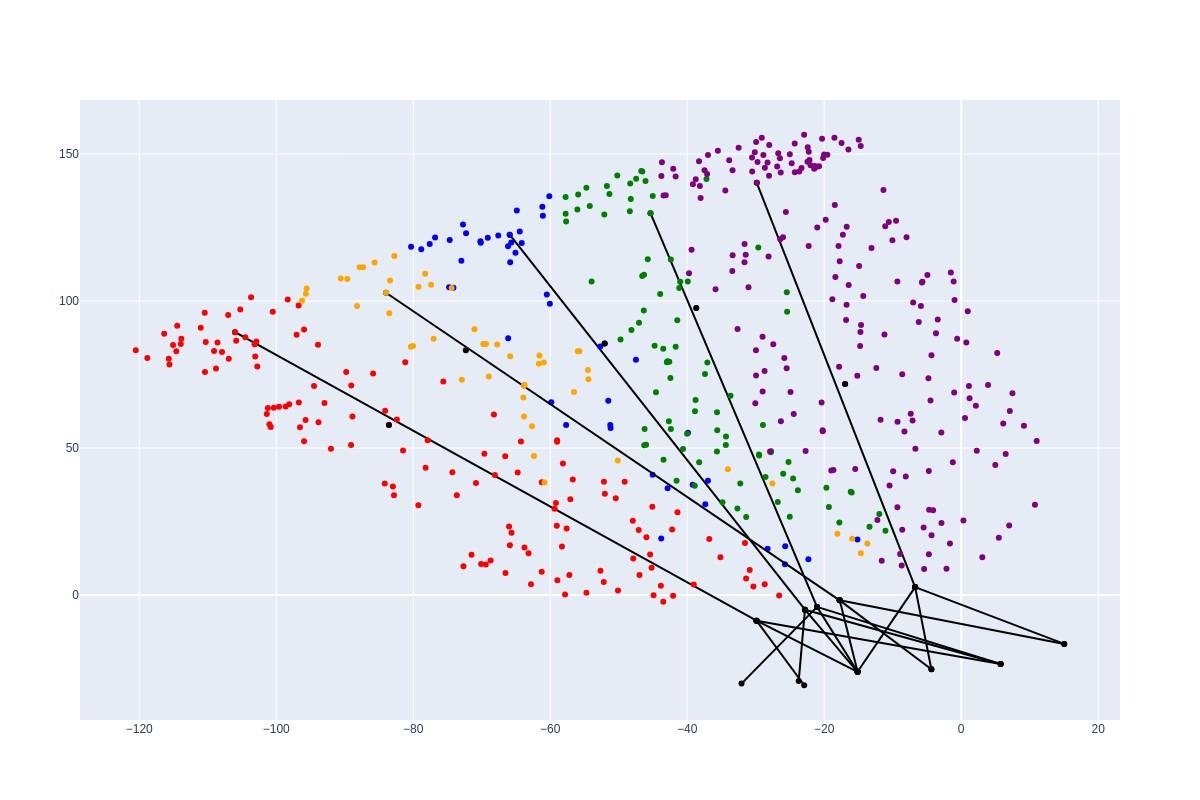


Appendix Figure 8: SOO Yarding Workload Objective


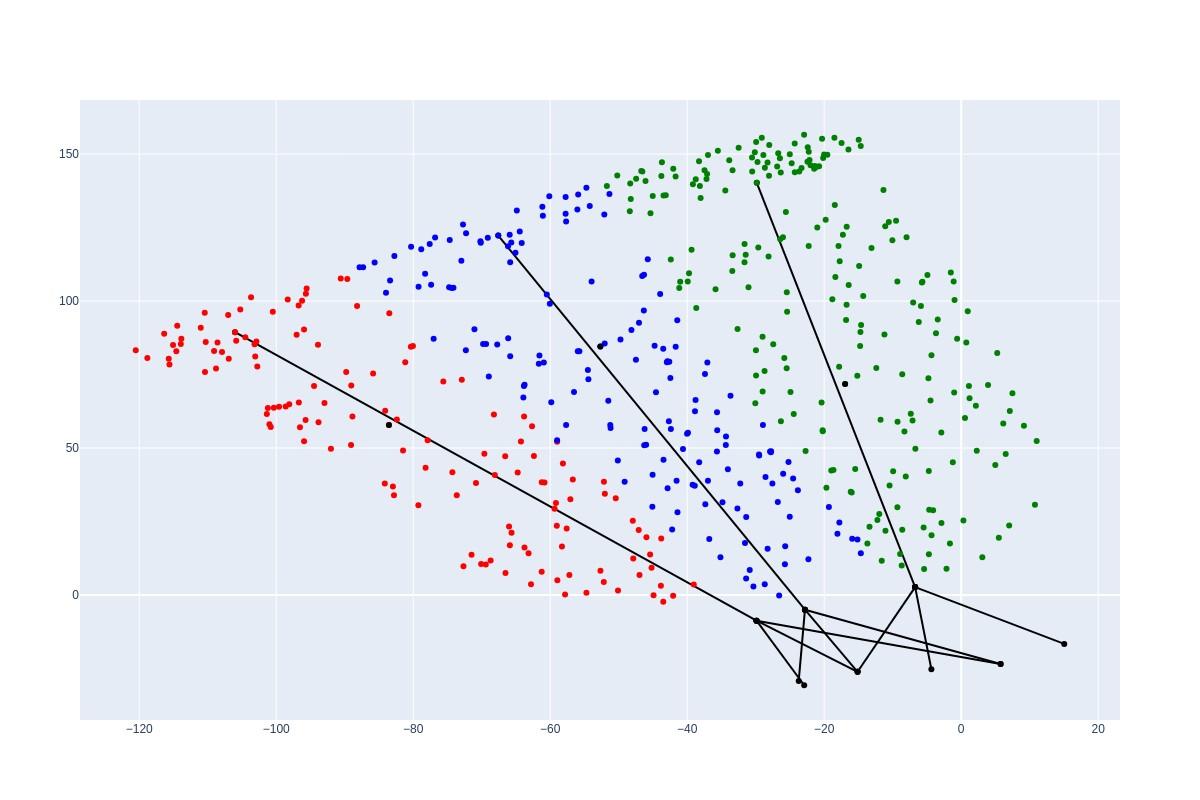


Appendix Figure 9: AUGMECON 00


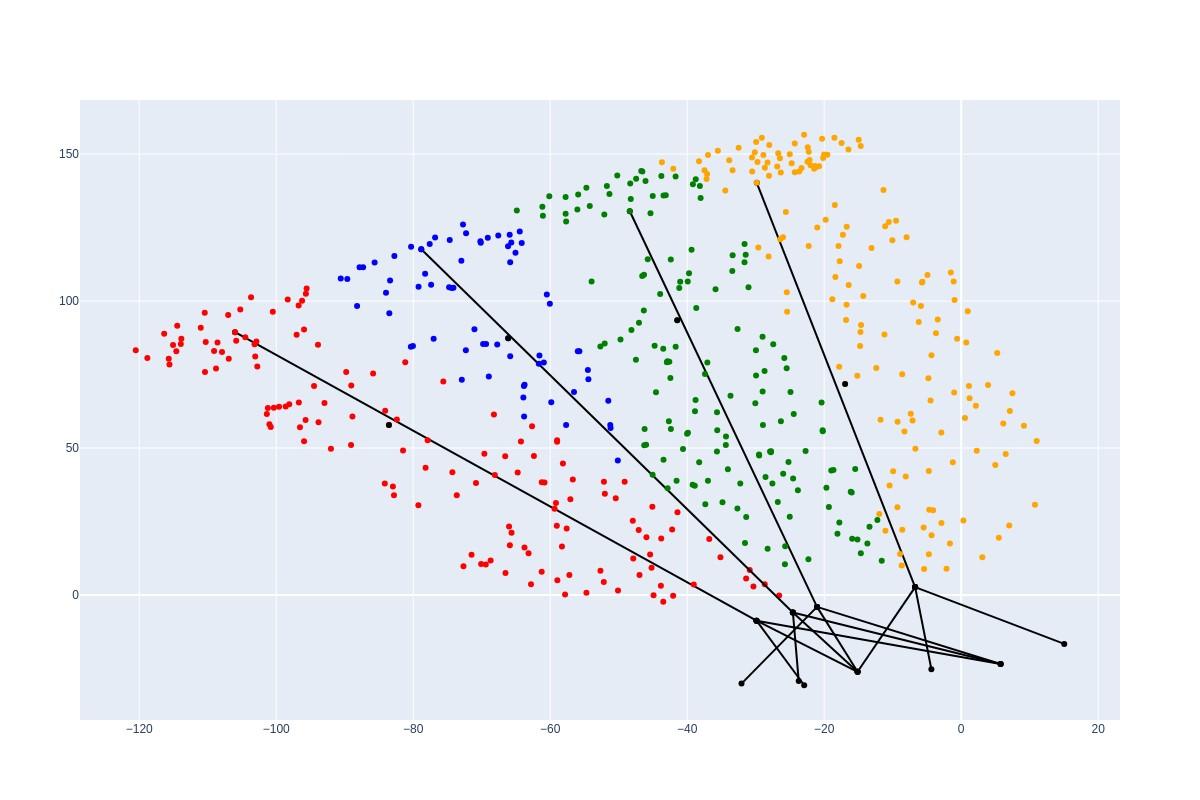


Appendix Figure 10: AUGMECON 04


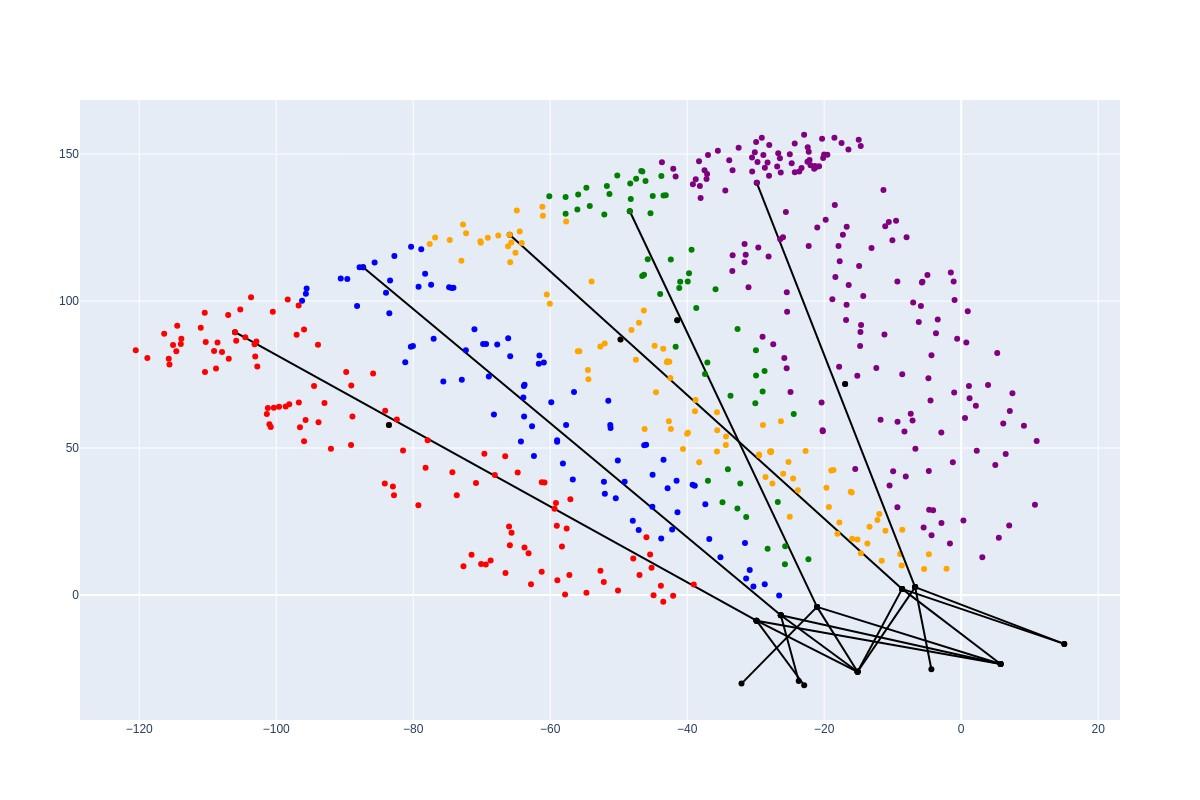


Appendix Figure 11: AUGMECON 40


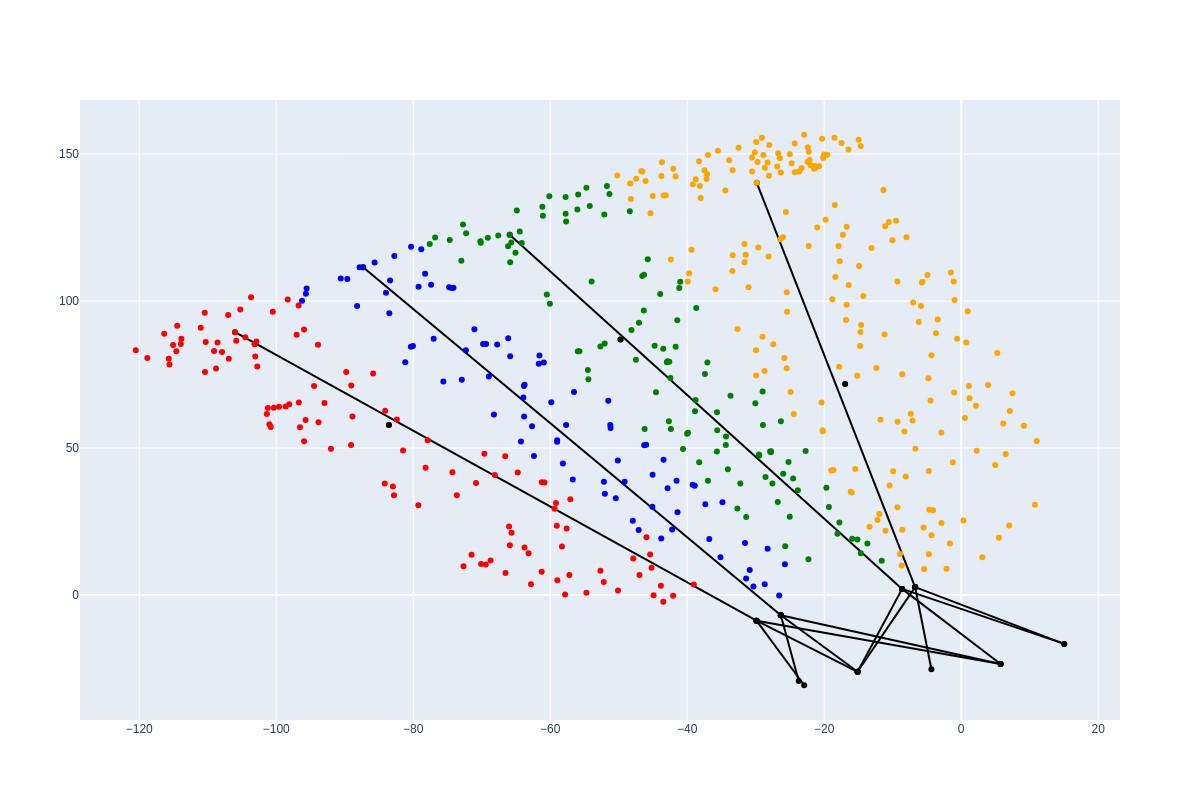


Appendix Figure 12: NSGA 3


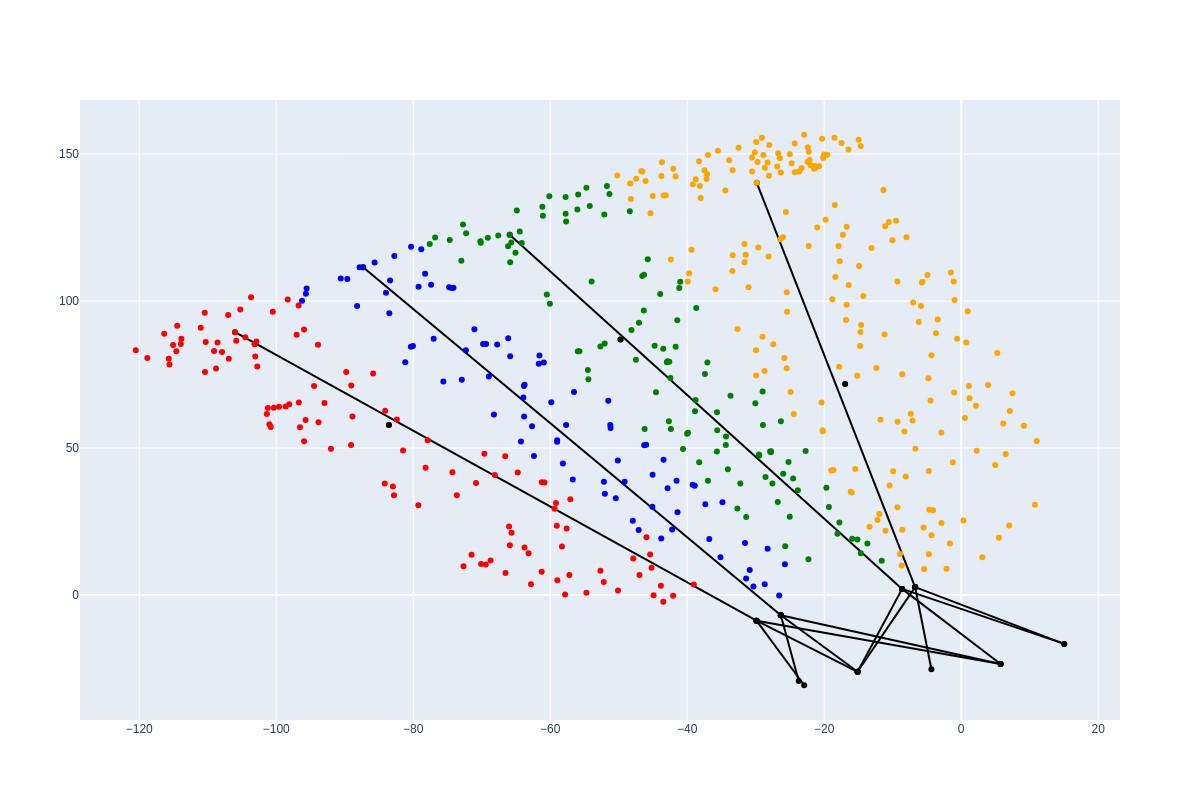


Appendix Figure 13: NSGA 0


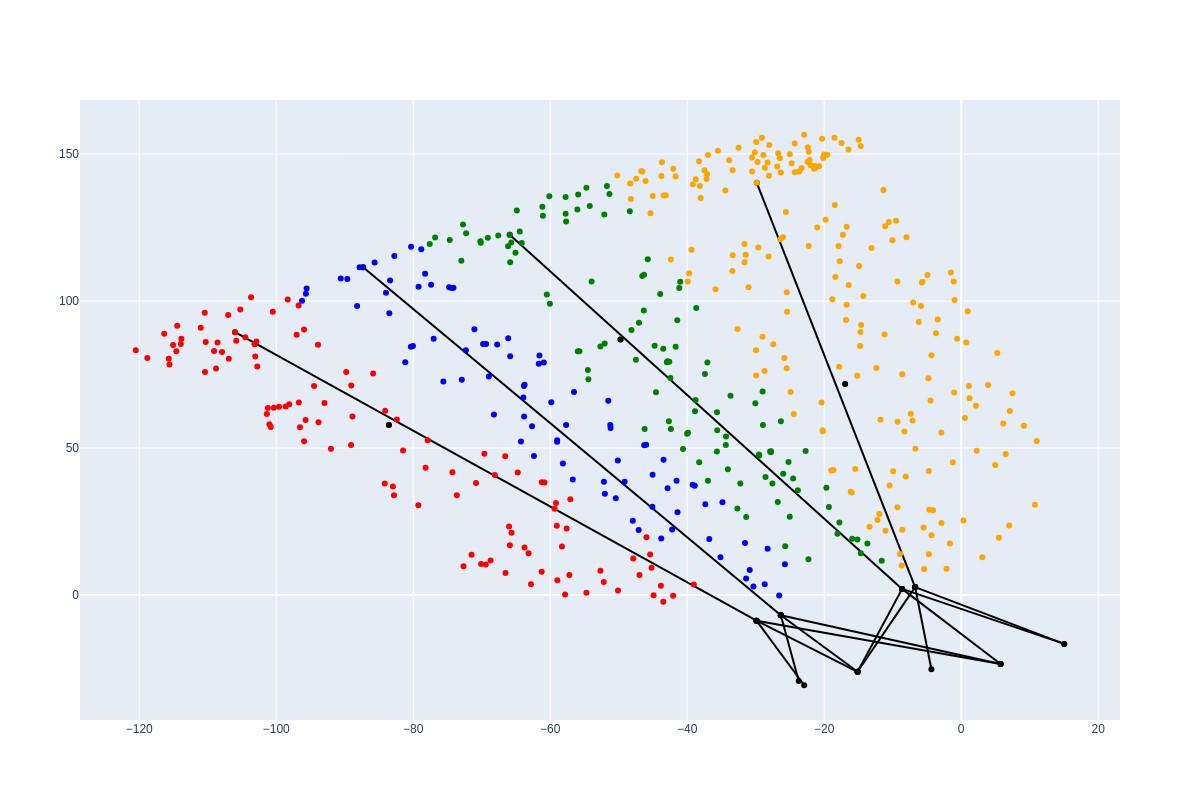


Appendix Figure 14: NSGA 6
